# Determining the Appropriate Support for Older Adults with Different Levels of Vitality and Health-Related Quality of Life: An Explanatory Study

**DOI:** 10.3390/ijerph20116052

**Published:** 2023-06-05

**Authors:** Damien S. E. Broekharst, Sjaak Bloem, Marije Blok, Mariët Raatgever, Nathascha Hanzen, Jasmien J. E. de Vette

**Affiliations:** 1 Center for Marketing & Supply Chain Management, Nyenrode Business University, 3621 BG Breukelen, The Netherlands; 2 Faculty of Social Sciences, Vrije Universiteit Amsterdam, 1081 HV Amsterdam, The Netherlands; 3 Center for Oncology, Bravis Hospital, 4708 AE Roosendaal, The Netherlands; 4 Janssen-Cilag B.V., Johnson & Johnson, 4837 DS Breda, The Netherlands

**Keywords:** subjective experienced health, vitality, health-related quality of life, older adults, elderly, ageing, healthy ageing

## Abstract

Vitality and health-related quality of life are often assessed in older adults. However, these assessments do not provide guidance on support for older adults with different levels of vitality and health-related quality of life. This guidance can be established through segmentation. The Subjective Health Experience model segments individuals and indicates support for each segment. By examining how older adults with different levels of vitality and health-related quality of life correspond with each segment and by specifying the indicated support to older adults, guidance can be established. This was examined by administering a questionnaire to 904 older adults and interviewing 8. Analysis was performed using one-way ANOVA and the matrix method. In segment 1, older adults sustained higher levels of vitality and health-related quality of life relative to other segments. They need information and certainty. In segment 2, older adults sustained lower levels of vitality and health-related quality of life relative to segment 1, and higher levels relative to segment 3 or 4. They need planning and structure. In segment 3, older adults sustained lower levels of vitality and health-related quality of life relative to segment 1 or 2, and higher levels relative to segment 4. They need emotive assistance. In segment 4, older adults sustained lower levels of vitality and health-related quality of life relative to other segments. They need personal coaching. As levels of vitality and health-related quality of life correspond with the segments, deploying vitality and health-related quality of life measures together with the model might be beneficial.

## 1. Introduction

**Figure 1:**
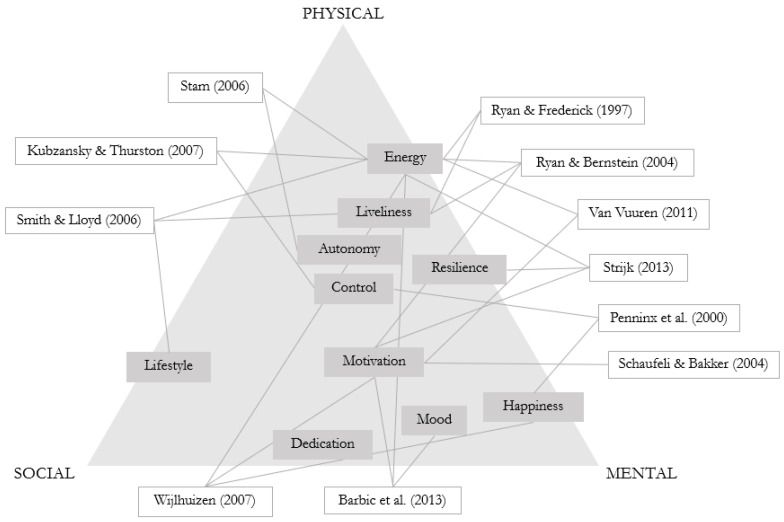
Different dimensions of vitality [9,10,11,12,13,14,15,16,17,18,19].

People worldwide are living longer as the average life expectancy has increased from 66.8 to 73.4 years over the last two decades [1]. Due to this ascending trend in life expectancy, the concept of healthy ageing has come to prominence in current healthcare practices [2]. The World Health Organisation (WHO) defines healthy ageing as the process of developing and maintaining the functional ability that enables wellbeing in older age [3]. In order to determine healthy ageing and ensure that people age healthily, general practitioners, nursing home physicians, geriatricians, mental health professionals, and others often assess the vitality and health-related quality of life of older adults [4,5]. The concept of vitality refers to an individual’s ability and motivation to autonomously sustain a lifestyle that enables him or her to live, grow, and develop in a vigorous, active, and lively manner [4,5,6]. The concept of vitality encapsulates a variety of dimensions (Figure 1), of which energy, motivation and resilience are most often associated with older adults [6,7]. The concept of health-related quality of life refers to an individual’s experience of physical and mental functioning while living his or her life the way he or she wants to, within the actual constraints and limitations of individual existence [8]. The concept of health-related quality of life can be identified as a multidimensional construct that spans several life domains or a unidimensional construct that considers the holistic nature of individual experience [8]. However, assessing vitality and health-related quality of life in older adults does not necessarily provide information and guidance on the appropriate support for older adults with different levels of vitality and health-related quality of life [8].

**Figure 2:**
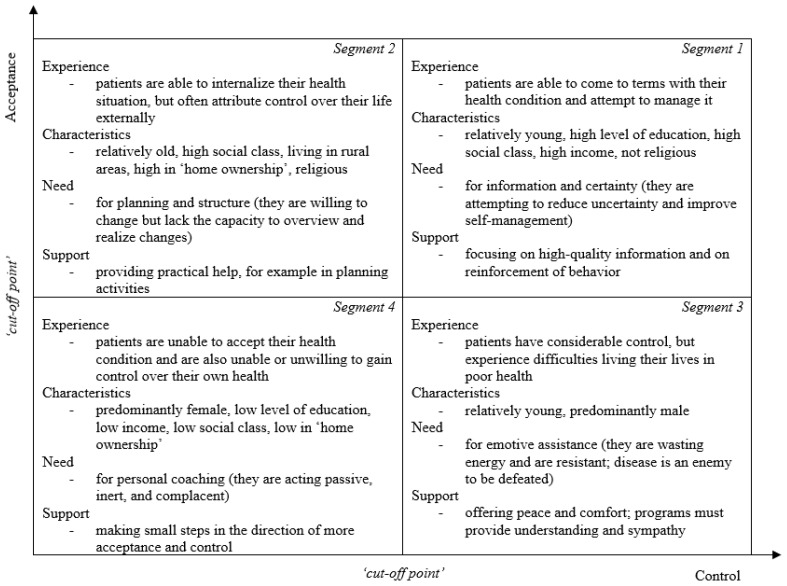
Subjective Health Experience model.

One method utilized to obtain an accurate indication concerning the appropriate support for older adults with different levels of vitality and health-related quality of life is segmentation [8,20]. An often-utilized segmentation model in healthcare is the Subjective Health Experience (SHE) model developed by Bloem et al. and extended by Broekharst et al. (Figure 2) [8,20]. This segmentation model constitutes four segments that each describe a particular subjective health experience profile, namely, health experience, population characteristics, healthcare needs, and appropriate support, based on the two central determinants of subjective health experience, namely, acceptance and control [8,20]. Individuals in segment 1 are able to come to terms with their health condition and attempt to manage it (high acceptance, high control) [8,20]. These individuals need high-quality information and reinforcement of desirable behavior (information and certainty) [8,20]. Individuals in segment 2 are able to internalize their health situation, but often attribute control over their life externally (high acceptance, low control) [8,20]. These individuals need practical assistance with regard to, for instance, planning activities (planning and structure) [8,20]. Individuals in segment 3 have considerable control, but experience difficulties living their lives in poor health (low acceptance, high control) [8,20]. These individuals need peace and comfort as well as understanding and sympathy (emotive assistance) [8,20]. Individuals in segment 4 are unable to accept their health condition and are also unable or unwilling to gain control over their own health (low acceptance, low control) [8,20]. These individuals need personal attention in order to make small steps in the direction of more acceptance and perceived control (personal coaching) [8,20].

In order to indicate the appropriate support for older adults with different levels of vitality and health-related quality of life, it is necessary to understand to which segment of the SHE model these particular individuals belong and specify the broadly defined appropriate support for individuals in the different segments to an older population. Therefore, this study examined: (1) how older adults with different levels of vitality and health-related quality of life correspond with different segments of the SHE model, and (2) how the appropriate support for individuals in different segments of the SHE model might be specified to an older population. The results of this study can be used by general practitioners, nursing home physicians, geriatricians, mental health professionals, and others in order to identify the appropriate support for older adults with different levels of vitality and health-related quality of life, assisting them in the process of healthy ageing.

## 2. Methods

### 2.1. Research Design

In this study, an explanatory sequential research design was used in which both qualitative and quantitative techniques were deployed [21,22,23]. An explanatory sequential research design involves the procedure of first gathering quantitative data in order to reveal general trends and patterns and then collecting qualitative research in order to help verify and elaborate upon the quantitative findings [21,22,23]. An explanatory sequential research design allows researchers to seek refinement, enhancement, elaboration, illustration, and clarification of findings from one method with the findings from another method [21,22,23].

### 2.2. Data Collection

#### 2.2.1. Questionnaire Study

In order to examine how older adults with different levels of vitality and health-related quality of life correspond with the different segments of the SHE model, a questionnaire study was conducted. For this study, a sample of 904 older adults (>60 years of age) was recruited from a panel of older adults who were involved in previous research of the Dutch National Foundation for Older Adults. Only respondents who formally consented and declared that they had no objection to their responses being used for research were included in this study. These respondents were also screened for duplicate panel memberships. The respondents were presented with six individual items in order to determine population characteristics, namely, gender, age, urbanization level, household size, education level, and living arrangement. These items were measured on nominal scales with dichotomous response categories or ordinal scales using ascending response categories. The respondents were subsequently presented with the Vita-16 questionnaire in order to assess 3 core dimensions of vitality, namely energy (5 items), motivation (6 items), and resilience (5 items) [6]. These items were measured on a quasimetric (interval) scale ranging from 1 = seldom to 7 = always [6]. The respondents were also presented with two health-related quality of life ladders on which instant (same day) and remembered (previous month) health and wellbeing were indicated [8]. These items were measured with an idiosyncratic and self-anchored visual ladder scale with 11 levels [8]. The respondents were further presented with the SHE model questionnaire in order to assess the determinants of subjective health experience, namely acceptance (3 items) and control (3 items) [8]. These items were measured on a quasimetric (interval) scale ranging from 1 = fully disagree to 7 = fully agree [8].

#### 2.2.2. Interview Study

In order to examine how the appropriate support for individuals in the different segments of the SHE model might be specified to an older population, an interview study was conducted. For this study, eight older adults (>60 years of age) were recruited from a panel of older adults who were involved in previous research of the Dutch National Foundation for Older Adults. This sample was deemed sufficient as this interview study was meant to provide the outcomes of the questionnaire study with additional detail and face validity. Only respondents who formally consented and declared that they had no objection to their interviews being used for research were included in this study. In the interview study the respondents were asked: (1) if they recognize themselves in the outcomes of the questionnaire study and the different segments of the SHE model, and (2) if they could further specify appropriate support for older adults with different levels of vitality and health-related quality of life in the different segments of the SHE model. The interviews were conducted based on a predefined topic list and had a duration of approximately 75 min. The interviews took place online via Microsoft Teams (Microsoft, Redmond, WA, USA) and the software program Mural (Mural, San Francisco, CA, USA) was deployed in order to increase interactivity. For instance, video excerpts were shown and Post-It notes with answers of the respondents were added to the screen during the interviews. The interviews were recorded, transcribed verbatim, and pseudonymized. None of the respondents received a monetary incentive.

### 2.3. Data Analysis

#### 2.3.1. Questionnaire Study

In order to assess the reliability of the different measures, Cronbach’s alpha coefficients were determined. The threshold for good reliability constitutes a Cronbach’s alpha coefficient of 0.70 (α > 0.70) [24,25,26,27]. In order to assess the validity of the different measures, factor analysis was conducted. After the factor analysis no items were removed. The segments of the SHE model were constructed by condensing the three items on acceptance and the three items on control into two independent scales that generated two single scores for every respondent [8]. The cut-off scores for the segments are 5.0 measured on a 7-point Likert scale and correspond with the medians of the distributions of acceptance and control, as was determined in previous work of Bloem et al. [8]. Based on the individual acceptance and control scores and the aforementioned cut-off scores, respondents were distributed over the four segments of the SHE model. In order to examine how older adults with different levels of vitality and health-related quality of life correspond with the different segments of the SHE model, a one-way ANOVA table was constructed and a Tukey test was conducted (α = 0.05) [28,29].

#### 2.3.2. Interview Study

The transcripts of the interviews were analyzed using the matrix method as this is a versatile method appropriate for uncovering meaningful patterns in interviews [30,31]. First, a raw matrix was created in which the interview questions represented the rows and the participants represented the columns. Second, the raw matrix was filled with the comments of the individual participants regarding specific interview questions. Third, a categorized matrix was created by establishing insight and charting common themes concerning different interview questions. Fourth, a tally matrix was established by tallying the occurrence of these common themes in order to create insight into their relative dominance. Fifth, based on the relative dominance of certain common themes, the appropriate support for older adults with different levels of vitality and health-related quality of life was identified.

## 3. Results

### 3.1. Population Characteristics

The sample for the questionnaire study showed that the distribution of gender, age, urbanization level, household size, and education level closely resembled the older population in the Netherlands (>60 years of age). However, it was also concluded that the sample represented a population that sustained a relatively independent living arrangement as compared to the older population in the Netherlands (>60 years of age). The population characteristics of the sample are displayed in Table 1.

The sample for the interview study consisted of six women and two men between the ages of 66 and 92 years. Although women generally have a higher life expectancy than men, they may still be slightly overrepresented in the sample. The questionnaires used in this study can be considered reliable as the scales on energy (α = 0.92), motivation (α = 0.95), resilience (α = 0.93), overall vitality (α = 0.88), acceptance (α = 0.91), and control (α = 0.91) all exceed the minimal Cronbach’s alpha coefficient threshold (α > 0.70).

### 3.2. Appropriate Support

**Figure 3:**
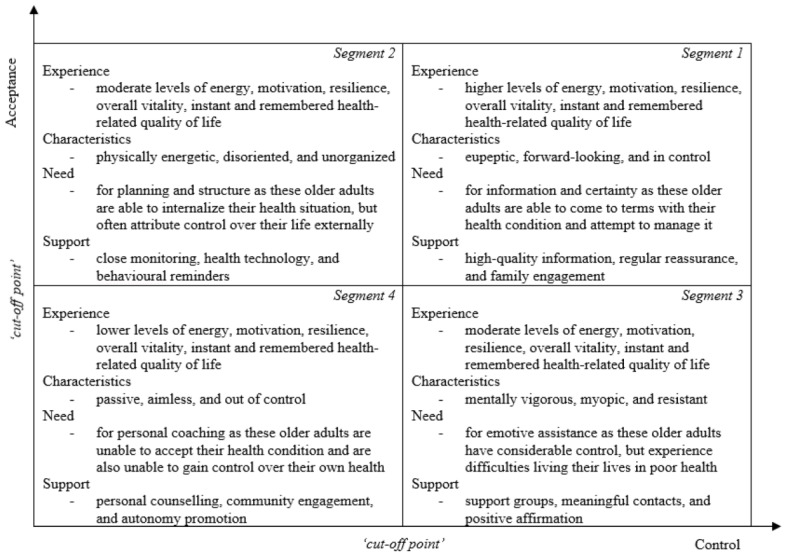
Subjective Health Experience model specified to older adults (preliminary).

The levels of energy [F(3, 842) = 185.005, *p* < 0.001], motivation [F(3, 817) = 82.262, *p* < 0.001], resilience [F(3, 820) = 93.847, *p* < 0.001], overall vitality [F(3, 769) = 146.453, *p* < 0.001], instant health-related quality of life [F(3, 852) = 98.671, *p* < 0.001], and remembered health-related quality of life [F(3, 845) = 85.011, *p* < 0.001] experienced by older adults significantly impacted the segment of the SHE model in which they are distributed and, subsequently, the support that is appropriate for them (Figure 3). The levels of energy, motivation, resilience, and overall vitality differ significantly between all segments, except for segment 2 and 3, in which only the level of energy significantly differs. The levels of instant and remembered health-related quality of life differ significantly between all segments.

#### 3.2.1. Segment 1

In total, 336 older adults (37.2%) were assigned to segment 1. The results show that the levels of energy (M = 5.33, SD = 1.11, 95% CI [5.21, 5.45]), motivation (M = 5.52, SD = 1.21, 95% CI [5.39, 5.66]), resilience (M = 4.64, SD = 0.853, 95% CI [4.55, 4.74]), overall vitality (M = 5.16, SD = 0.95, 95% CI [5.05, 5.27]), instant health-related quality of life (M = 7.84, SD = 1.30, 95% CI [7.70, 7.99]), and remembered health-related quality of life (M = 7.46, SD = 1.30, 95% CI [7.31, 7.61]) among older adults assigned to segment 1 (high acceptance, high control) are higher than among those assigned to segments 2, 3, and 4. As older adults with these levels of vitality and health-related quality of life correspond with segment 1 of the SHE model, they often need information and certainty (Figure 2). The interviewees agreed that older adults corresponding with segment 1 are in need of information and certainty as these older adults might be considered in control, eupeptic, and forward-looking. They argued that relatively young older adults may be more forward-looking than those that are older. The interviewees further explained that older adults with a need for information and certainty may specifically require support in terms of (1) high-quality information on their health state, (2) regular reassurance by a healthcare professional, and (3) family backup to promote self-efficacy (Figure 3). 

#### 3.2.2. Segment 2

In total, 169 older adults (18.7%) were assigned to segment 2. The results subsequently indicate that the levels of energy (M = 4.39, SD = 1.25, 95% CI [4.20, 4.59]), motivation (M = 4.91, SD = 1.29, 95% CI [4.71, 5.12]), resilience (M = 4.11, SD = 0.92, 95% CI [3.97, 4.26]), overall vitality (M = 4.47, SD = 0.95, 95% CI [4.31, 4.62]), instant health-related quality of life (M = 7.37, SD = 1.39, 95% CI [7.16, 7.59]), and remembered health-related quality of life (M = 6.96, SD = 1.35, 95% CI [6.75, 7.17]) among older adults assigned to segment 2 (high acceptance, low control) are lower than among those assigned to segment 1, and higher than among those assigned to segment 3 or 4. As older adults with these levels of vitality and health-related quality of life correspond with segment 2 of the SHE model, they often need planning and structure (Figure 2). The interviewees confirmed that older adults corresponding with segment 2 are in need of planning and structure as these older adults might be considered disoriented, undisciplined, and physically energetic. They argued that relatively young older adults may be more physically energetic than those that are older. The interviewees further explicated that older adults with a need for planning and structure may specifically require support in terms of (1) close monitoring by healthcare professionals, (2) health technology to self-manage their health state, and (3) behavioral reminders to maintain healthy routines (Figure 3).

#### 3.2.3. Segment 3

In total, 52 older adults (5.8%) were assigned to segment 3. The results also indicate that the levels of energy (M = 4.01, SD = 1.04, 95% CI [3.70, 4.32]), motivation (M = 4.55, SD = 1.41, 95% CI [4.13, 4.97]), resilience (M = 3.84, SD = 1.10, 95% CI [3.52, 4.17]), overall vitality (M = 4.12, SD = 0.94, 95% CI [3.83, 4.41]), instant health-related quality of life (M = 6.78, SD = 1.28, 95% CI [6.41, 7.14]), and remembered health-related quality of life (M = 6.21, SD = 1.49, 95% CI [5.78, 6.64]) among older adults assigned to segment 3 (low acceptance, high control) are lower than among those assigned to segments 1 or 2, and higher than among those assigned to segment 4. As older adults with these levels of vitality and health-related quality of life correspond with segment 3 of the SHE model, they often need emotive support (Figure 2). The interviewees corroborated that older adults corresponding with segment 3 are in need of emotive assistance as these older adults might be considered mentally vigorous, myopic, and resistant. They argued that relatively young older adults may be more resistant than those that are older. The interviewees further clarified that older adults with a need for emotive assistance may specifically require support in terms of (1) support groups to find solace with peers, (2) meaningful contacts with significant others, and (3) positive affirmation from health professionals (Figure 3).

#### 3.2.4. Segment 4

In total, 347 older adults (38.4%) were assigned to segment 4. The results further indicate that the levels of energy (M = 3.09, SD = 1.30, 95% CI [2.95, 3.23]), motivation (M = 3.85, SD = 1.46, 95% CI [3.69, 4.01]), resilience (M = 3.30, SD = 1.13, 95% CI [3.18, 3.43]), overall vitality (M = 3.39, SD = 1.14, 95% CI [3.26, 3.52]), instant health-related quality of life (M = 5.81, SD = 1.85, 95% CI [5.61, 6.01]), and remembered health-related quality of life (M = 5.64, SD = 1.69, 95% CI [5.45, 5.82]) among older adults assigned to segment 4 (low acceptance, low control) are lower than among those assigned to segments 1, 2, and 3. As older adults with these levels of vitality and health-related quality of life correspond with segment 4 of the SHE model, they often need personal coaching (Figure 2). The interviewees concurred that older adults corresponding with segment 4 are in need of personal coaching as these older adults might be considered out of control, aimless, and passive. They argued that relatively young older adults may be less passive than those that are older. They further delineated that older adults with a need for personal coaching may specifically require support in terms of (1) personal counseling by healthcare professionals, (2) community engagement to prevent social isolation, and (3) assistance in maintaining personal autonomy (Figure 3).

## 4. Discussion

This study examines how older adults with different levels of vitality and health-related quality of life correspond with different segments of the SHE model and how the appropriate support for individuals in different segments of the SHE model may be specified to an older population. In segment 1, older adults sustained higher levels of vitality and health-related quality of life relative to segments 2, 3, or 4. These older adults often need information and certainty (high-quality information, regular reassurance, family backup). In segment 2, older adults sustained lower levels of vitality and health-related quality of life relative to segment 1 and higher levels relative to segments 3 or 4. These older adults often need planning and structure (structural monitoring, health technology, behavioral reminders). In segment 3, older adults sustained lower levels of vitality and health-related quality of life relative to segments 1 or 2 and higher levels relative to segment 4. These older adults often need emotive assistance (support groups, positive affirmation, meaningful contacts). In segment 4, older adults sustained lower levels of vitality and health-related quality of life relative to segments 1, 2, or 3. These older adults often need personal coaching (personal counseling, community engagement, autonomy promotion).

Previous research shows that older adults with higher levels of acceptance and control (segment 1) often sustain higher levels of vitality and health-related quality of life, which is in line with the results of this study [32,33]. In addition, this study suggests that these older adults often need information and certainty [8,20]. Previous research shows that older adults with a need for information and certainty often require support in terms of high-quality information and regular reassurance, which is in line with the findings of this study [34,35]. In addition, this study suggests that these older adults may also require support in terms of family backup as their assistance could instill self-efficacy in older adults. Preceding studies subsequently show that older adults with varying levels of acceptance and control (segments 2 and 3) often sustain moderate levels of vitality and health-related quality of life, which is in accordance with this study [32,33]. In addition, this study suggests that these older adults often need planning and structure or emotive assistance [8,20]. Preceding studies show that older adults with a need for planning and structure often require support in terms of structural monitoring and behavioral reminders, which is in concordance with this study [36,37]. In addition, this study shows that these older adults may also require support in terms of health technology as these technologies could aid older adults in self-managing their health state. Preceding studies show that older adults with a need for emotive assistance often require support in terms of support groups and meaningful contacts with significant others, which is in accordance with this study [38,39]. In addition, this study shows that these older adults may also require support in terms of positive affirmation as they may lack hope and meaning in their lives. Prior research also shows that older adults with lower levels of acceptance and control (segment 4) often sustain lower levels of vitality and health-related quality of life, which is in concordance with the results of this study [32,33]. In addition, this study suggests that these older adults often need personal coaching [8,20]. Prior research shows that older adults with a need for personal coaching often require support in terms of personal counseling and autonomy promotion, which is in concordance with this study [40,41]. In addition, this study shows that these older adults may also require support in terms of community engagement as volunteers and neighbors could prevent social isolation.

Finally, some further observations and considerations drawn from this study may be prudent to contemplate and discuss. It should be stipulated that the information and guidance on the appropriate support for older adults with different levels of vitality and health-related quality of life as provided by the different segments of the specified SHE model (Figure 3) is advisory in nature, making additional interpretation by general practitioners, nursing home physicians, geriatricians, mental health professionals, and others desirable. It should also be noted that it may be beneficial to deploy the SHE model questionnaire in combination with often-deployed vitality questionnaires and health-related quality of life ladders, as almost all of these measures lack information and guidance on the appropriate support for older adults with different levels of vitality and health-related quality of life [8]. It should further be remarked that it may be recommendable to use health-related quality of life ladders instead of vitality questionnaires as this study shows that the former are able to differentiate between segments 2 or 3, which the latter are not. This difference may be caused due to the relatively limited sensitivity of the vitality questionnaire compared to the health-related quality of life ladders [42].

### 4.1. Strengths and Limitations

An important strength of this study is that it utilized an explanatory sequential research design in which both qualitative and quantitative methods were combined in order to establish additional in-depth comprehension of the particular topic. Another important strength of this study is the considerable sample size that was obtained in the questionnaire study, thereby increasing the probability of representative and generalizable results. A subsequent limitation of this study relates to the apparent bias in the sample of the questionnaire study towards independently living older adults, thereby increasing the probability of skewed and distorted results. Another limitation of this study relates to the somewhat limited sample size that was obtained in the interview study, thereby increasing the probability of insufficient and incomplete results. A final limitation of this study relates to the slight bias in the sample of the interview study towards women, thereby increasing the probability of skewed and distorted results.

### 4.2. Future Research

There are, at least, three avenues for future research that may be considered based on this study. A first avenue for future research relates to repeating this research in different or stratified populations (e.g., men, women, healthy, sick, young, old) in order to examine the appropriate support for these populations. A second avenue for future research relates to specifying the broadly defined and rather generic support requirements described in the different segments of the SHE model to these different or stratified populations. A third avenue for future research relates to repeating this research with other often-measured concepts (e.g., autonomy, mood, lifestyle) in order to examine the appropriate support that corresponds with different levels of these particular concepts.

## 5. Conclusions

Given the results of this study, it is evident that the levels of vitality and health-related quality of life among older adults correspond with the different segments of the SHE model, providing general practitioners, nursing home physicians, geriatricians, mental health professionals, and others with information and guidance on the appropriate support for these older adults. Therefore, it may be beneficial to deploy the many often-used vitality questionnaires or health-related quality of life ladders in combination with the SHE model in order to not only assess vitality and health-related quality of life among older adults, but also to obtain information and guidance on the appropriate support for older adults with different levels of vitality and health-related quality of life.

## Figures and Tables

**Table 1:** Sample description.

Age	x̅ 72.7 Years (60–95 Years)			
Gender	38.3% male	61.7% female		
Urbanization level	66.0% city	31.6% rural area	2.4% unknown	
Household size	45.5% one-person	51.8% two-person	2.7% ≥ three-person	
Education level	3.3% primary	45.3% secondary	36.6% vocational	14.8% university
Living arrangement	47.1% independent (no partner)	51.4% independent (with partner)	0.4% care institution	1.1% other

## Data Availability

The dataset used during this study is not publicly available, but is available from the corresponding author upon reasonable request.
